# 4294 Patient Matching Errors and Associated Safety Events

**DOI:** 10.1017/cts.2020.160

**Published:** 2020-07-29

**Authors:** Melody Lynn Greer

**Affiliations:** 1 University of Arkansas for Medical Sciences

## Abstract

OBJECTIVES/GOALS: Errors in patient matching could result in serious adverse safety events. Unlike publicized mix-ups by healthcare providers these errors are insidious and with increased data sharing, this is a growing concern in healthcare. The following project will examine patient matching errors and quantify their association with safety. METHODS/STUDY POPULATION: EHR systems perform matching out-of-the-box with unknown quality. Using matching processes outside the EMR, the rate at which matching errors are present was quantified and the erroneous records were flagged providing both comparative measures and data necessary to evaluate patient safety. To understand the relationship between matching and safety we will establish a percent of voluntarily reported safety events in our institution where a matching error existed during an encounter. Any safety events occurring for a flagged patient will be reviewed to determine if matching errors contributed to the safety problem. Not all safety events are reported so we will perform full chart review of a filtered list of medical records that have a higher likelihood of safety events. RESULTS/ANTICIPATED RESULTS: We were able to quantify matching errors, and the preliminary matching error rate is approximately 1%, representing over 700 patients. The work is in progress and we are beginning to determine the association between safety events and incorrect matching. Together these results will provide an incentive to identify errors, make corrections, and develop methods to achieve these objectives. The number of matching errors impacts patient care as well as business operations and is likely to have a negative financial impact on institutions with high error rates regardless of its relationship to safety. DISCUSSION/SIGNIFICANCE OF IMPACT: Patient matching is bundled with EHR software and institutions have little control over error rates, yet bear the liability for resulting clinical error. Institutions need to be able to identify undetected matching errors and any associated safety events and this project will provide that solution.

